# The Scandinavian Solutions for Wellness study - a two-arm observational study on the effectiveness of lifestyle intervention on subjective well-being and weight among persons with psychiatric disorders

**DOI:** 10.1186/1471-244X-10-42

**Published:** 2010-06-10

**Authors:** Vibeke Porsdal, Catherine Beal, Ole Kristian Kleivenes, Egil W Martinsen, Eva Lindström, Harriet Nilsson, Pär Svanborg

**Affiliations:** 1 Eli Lilly Danmark A/S, Nybrovej 110, DK - 2800 Lyngby, Denmark; 2 Keyrus BioPharma, 53, rue Baudin, F-92300 Levallois-Perret, France; 3 Eli Lilly Norge A.S, Grenseveien 99, P.O. box 6090 Etterstad, N-0601 Oslo, Norway; 4 Clinic of psychiatric health, Aker University Hospital, Oslo, Norway; 5 Clinic of forensic psychiatry, Malmö University Hospital, Malmö, Sweden; 6 ICON Clinical Research Limited, Gårdsvägen 18, SE-169 27 Solna, Sweden; 7 Eli Lilly Sweden AB, Karolinska Institutet, Dept of Clinical Neuroscience, Stockholm, Sweden

## Abstract

**Background:**

Solutions for Wellness (SfW) is an educational 3-month program concerning nutrition and exercise for persons with psychiatric disorders on psychotropic medication, who have weight problems. This observational study assessed the impact of SfW on subjective well-being, weight and waist circumference (WC).

**Methods:**

Data was collected at 49 psychiatric clinics. Where the SfW program was offered patients could enter the intervention group; where not, the control group. Subjective well-being was measured by the Subjective Well-being under Neuroleptics scale (SWN), at baseline, at the end of SfW participation, and at a follow-up 6 months after baseline. Demographic, disease and treatment data was also collected.

**Results:**

314 patients enrolled in the SfW group, 59 in the control group. 54% of the patients had schizophrenia, 67% received atypical antipsychotics, 56% were female. They averaged 41 ± 12.06 years and had a BMI of 31.4 ± 6.35. There were significant differences at baseline between groups for weight, SWN total score and other factors. Stepwise logistic models controlling for baseline covariates yielded an adjusted non-significant association between SfW program participation and response in subjective well-being (SWN increase). However, statistically significant associations were found between program participation and weight-response (weight loss or gain < 1 kg) OR = 2; 95% CI [1.1; 3.7] and between program participation and WC-response (WC decrease or increase < 2 cm) OR = 5; 95% CI [2.4; 10.3]), at 3 months after baseline.

**Conclusions:**

SfW program participation was associated with maintaining or decreasing weight and WC but not with improved subjective well-being as measured with the SWN scale.

## Background

Weight gain and consequent obesity is a serious and growing health problem, associated with an increased risk of a number of somatic illnesses, e.g., type II diabetes and cardiovascular disorders and with a reduction in quality of life [1-3]. Persons with schizophrenia are even more prone to weight gain and obesity than the general population; possible reasons for this include change in caloric intake and physical inactivity [4]. In addition to this, weight gain is an adverse effect associated with many antipsychotics, antidepressants and mood stabilizers [4-8].

Several studies have investigated whether behavioral interventions can be effective in curbing the weight gain induced by psychotropic medication [9-24]. The intervention programs evaluated in these studies ranged from comprehensive group programs with motivational counseling, physical exercises and teaching healthy eating or with cognitive therapy focusing on cognition in relation to food and body weight to educational mailings about the importance of diet and exercise. The designs of the studies were diverse, encompassing single-arm observational studies, studies with matched controls and randomized controlled trials. In the recent years, a few reviews of studies of behavioural weight management in persons with serious mental illness have been made [25-28]. These reviews concurrently conclude that it is possible to manage weight gain in persons with serious mental illness by means of behavioral interventions.

Only a few of the studies mentioned above took the next step and included evaluation of the impact of behavioural weight management on the quality of life (QoL) or subjective well-being of the participants. In one randomized trial, a one-on-one nutrition education program was found to increase QoL and reduce weight gain [18]. Another randomized trial evaluating an individual program focusing on diet and exercise found that the persons attending the program reduced the weight more than the controls, but no difference was found between the groups for QoL [10]. A third randomized trial evaluated a group program comprising diet and physical activity [24]. This program was also found to reduce weight, but QoL was not improved. A single-arm study found decreased weight and improved QoL after a group program comprising teaching healthy lifestyles as well as physical exercise [9].

The Solutions for Wellness (SfW) educational program in Scandinavia (Denmark, Norway and Sweden) is a well-established 12-week group program for persons who suffer from psychiatric disorders, use psychotropic medications and have weight problems. The program focuses on working towards a healthier lifestyle, with two main elements: healthier diet and increased physical activity. The primary objective of the observational study presented here was to investigate the impact of the Scandinavian SfW educational program on the development of subjective well-being and QoL as measured by changes in the Subjective Well-Being under Neuroleptics (SWN) scale. The population studied was suffering from chronic psychiatric illness and treated by any psychotropic medication. Secondary objectives included to assess if potential changes in subjective well-being and QoL during the program were maintained at a follow-up 3 months after the program ended, to describe the association between participants' characteristics at baseline and SWN changes during the program and 3 months later, and finally to describe development in weight, BMI and waist circumference after completion of the program and 3 months later, as well as potential associations with baseline variables.

## Methods

### Study design

This observational study was carried out at 49 sites, 42 Solutions for Wellness (SfW) sites and 7 control sites, located in Denmark, Norway and Sweden from 9-Aug-2006 to 28-Feb-2008. The SfW sites included psychiatric departments and clinics where the SfW program was well established in normal clinical practice. Persons about to enter the program were invited to enroll in the study. The control sites were recruited among psychiatric departments and clinics that had not initiated the SfW program yet. The control sites invited persons, who met the study eligibility criteria, to enroll in the study as the control group.

Eligible persons were either outpatients or inpatients in non-acute wards (i.e., patients whose condition was relatively stable). Criteria for enrollment included having a psychiatric diagnosis and being on psychotropic medication, defined as antipsychotics, antidepressants or mood stabilizers. They should also have a weight problem (being overweight or gaining weight) in their personal opinion and in the judgment of their healthcare provider, but should not be on any pharmacologic intervention for weight gain. Ethical review boards and regulatory authorities' approval were obtained in compliance with local laws, and a written informed consent was given by each person before entering the study.

The study consisted of 3 visits, where the baseline visit was equivalent to the first lesson of the SfW program. The endpoint visit occurred 3 months after baseline and was equivalent to the last lesson or lesson 12 in case the program was longer than 12 weeks. The follow-up visit took place 6 months after baseline, i.e., 3 months after the SfW program completion. The control group participants only had two data collection points, baseline and endpoint (3 months apart). Decisions on the treatment and care were made by the healthcare providers according to normal clinical practice, and collected data reflect real-life practice.

### Solutions for Wellness educational program

"Solutions for Wellness" has different meanings in different countries. In Scandinavia, SfW is a standardized program, which is performed in a group setting in psychiatric departments and clinics under the guidance of specially trained nurses or therapists. The specially trained nurses or therapists are health care professionals who have their daily work in the participating departments and clinics, and who have been trained by the same, few SfW project managers, individually and in group trainings. The SfW program in Scandinavia usually consists of 12 lessons that focus on nutrition/eating habits or physical activity or both of these subjects. The lessons are based on written material (manuals for patients and program coordinators). The written material covers 18 subjects related to eating and 14 subjects related to physical activity, and the nurses and therapists select and combine these subjects according to the needs of the program participants. The lessons are given to small groups of patients (often 4-8), who will go through the program together, typically with one lesson per week.

### Assessment of outcome

At each visit, weight and waist circumference were measured and the participants assessed their subjective well-being by answering the questions in the Subjective Well-being under Neuroleptics scale (SWN) [29,30] and with a generic QoL instrument, 15D [31]. The investigators rated the Clinical Global Impression of severity (CGI-S) at all visits [32]. Participants were weighed at the same time of the day on the same scale without wearing shoes.

Waist circumference was measured between the lowest rib margin and the iliac crest. All study procedures, except for the self-assessed 15D QoL questionnaire and the investigators' assessment of CGI-S, were integrated parts of the SfW program.

Information on demographics (age, sex and height), medical history (diagnosis, duration of illness) and psychotropic medication at entry into the program was collected at the baseline visit. Number of SfW lessons attended was registered at the endpoint visit.

### Health outcome/quality of life measures

The **Subjective Well-Being under Neuroleptics (SWN) **is a self-rating scale constructed by Naber and coworkers, [29,33] with the underlying assumption that subjective well-being is a major determinant of medication compliance in schizophrenia. Neuroleptic drugs differ with respect to their tendency to produce side effects, and the SWN scale was developed to cover such differences in side effects, resulting in five subscales: Mental functioning, Social integration, Emotional regulation, Physical functioning and Self-control. The SWN scale is only moderately correlated to objective psychopathology as assessed with the positive and negative syndrome scale (PANSS) and to the established Udvalg for kliniske undersøgelser (UKU) side effect scale [30,34]. The original SWN scale consists of 38 items and has been used in several international trials [30]. In order to improve the utility of the instrument, a short form was developed, consisting of 20 items with four items covering each of the five dimensions. The short form has good psychometric properties [30,35] and it was used in the present study. Items are scored on a 1 - 6 scale, where 1 is indicating "not at all" and 6 is indicating "very much". Total score and subscales scores were computed.

The **15D Quality of Life **instrument is a generic health-related QoL instrument that, due to its simplicity and easy applicability, can always be included in health program evaluation studies [31]. The 15D covers 15 items, namely: mobility, vision, hearing, breathing, sleeping, eating, speech, elimination, usual activities, mental function, discomfort and symptoms, depression, distress, vitality and sexual activity. Each item comprises one question with five answer options. A single index score on a 0-1 scale (0 = being dead, 1 = full health) is computed. The 15D has been used in projects evaluating medical interventions and rehabilitation and in National Health surveys in Finland and Denmark. Based on empirical research, a change of 0.02-0.03 on the single index score of 15D is considered clinically relevant [31].

The **Clinical Global Impression - Severity (CGI-S) **is a single-item clinician rating of the severity of the person's psychiatric symptoms in relation to the clinician's total experience of previous persons with the same diagnosis [32]. Severity is rated on a seven-point Likert scale (1 = normal, not ill at all, 7 = among the most extremely ill patients).

### Determination of sample size

The primary objective of this study was to describe the participants' change in subjective well-being, as measured by the SWN, at the end of the three-month period for the SfW group compared to the control group. No clinically meaningful change has been defined for the SWN scale before, and it was decided to use a between-group difference in the proportion of participants with any SWN improvement for the sample size calculation. The sample size was estimated based on the following assumptions and settings: an expected proportion of participants with any SWN improvement of 50% in the control group and of 70% in the SfW group, a power of 80% to detect such a difference and a two-sided significance level of 0.05. Enrollment of 300 participants was planned for the SfW group and 55 participants for the control group, and enrollment ended when baseline and endpoint observations had been registered from these numbers of persons.

### Statistical analysis

Analyses were conducted in the full analysis set (all enrolled persons). Missing data were not replaced, for each SWN-subscale, a missing answer to any item yielded a missing sub-score and, therefore a missing total score. All baseline characteristics were summarized using standard descriptive statistics by study group (SfW/control) and, for diagnosis-related baseline data (collected at visit 1), by diagnosis. Baseline variables were compared between groups by means of the chi-square or the Fisher-Freeman-Halton exact test for categorical variables, and the t-test in case of continuous variables. Changes over time within each group were tested by means of the paired t-test.

The primary analysis tested the between-group difference on the rate of responses in terms of subjective well-being at completion of the program (percentage of subjects who experienced an improvement, i.e. SWN increase after 3 months), using the Chi-square test. The response rates at month 3 were estimated in the SfW and control groups, as well as their between-group difference and 95% confidence intervals (exacts). At month 3, stepwise logistic regression models were fit to assess the SfW association with subjective well-being. A logit-link function was chosen to relate the dichotomous response variable of improving subjective well-being adjusting for the following baseline variables: gender, age, psychiatric diagnosis (bipolar, schizophrenia, other), duration of illness, and symptom severity (CGI-S scale), SWN score, waist measurement, weight, BMI and psychotropic drugs. The significance level for entry in the model was 0.10 and 0.05 for remaining in the model. First degree interactions between main effects still present were tested through a second stepwise process using the same significance levels as above. The group variable was forced in the model and maintained even if not significant. One-by-one interactions were fitted into the model as well and maintained if significant.

As a measure of association between the response variable and the independent variables, the odds ratios (ORs) with the relative 95% confidence interval as well as the p-value, were reported. For continuous variables, to provide a useful interpretation, the estimated ORs were expressed for a change of 1 unit in the covariate.

A similar analysis, conducted only in the SfW group, was conducted on the response at month 6, replacing the study group by the number of SfW lessons attended. Crude mean changes from baseline to 3 months and 6 months, as well as from 3 months to 6 months, in SWN scores/subscores, weight, BMI and waist circumference were estimated along with their 95% confidence intervals.

A sensitivity analysis was conducted after implausible waist circumference values were discovered lately in the database (changes from 25 cm to 96 cm over 3 months) to assess their impact on results (impossible values were set to missing).

A post-hoc analysis was performed to further investigate the response rates for weight, waist circumference and CGI-Severity, as well as associated factors. For waist circumference a decrease was defined as a decrease of at least 2 cm, a stable waist circumference was defined as a change between - 2 cm and 2 cm, and an increase was defined as 2 cm increase or more. For weight a decrease was defined as a loss of at least 1 kg, stable weight was a change between - 1 and 1 kg, and an increase was a gain of at least 1 kg. For waist circumference and weight the response was defined as a decrease or stable waist circumference or weight, as described above. For CGI-S, the response was defined as a decrease. All analyses were computed using SAS^®^9, SAS Institute Inc., SAS Campus Drive, Cary, North Carolina 27513, USA.

## Results

### Participant characteristics

**Figure 1:**
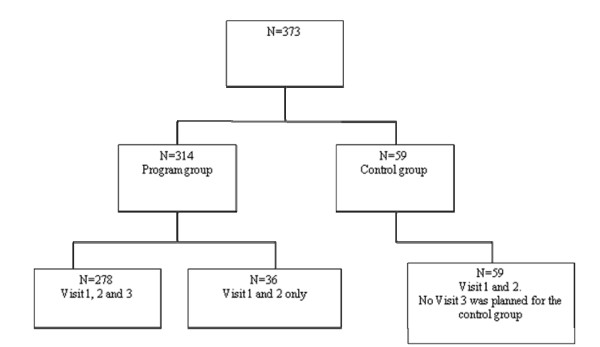
**Participant flow**.

In total, 373 participants were enrolled in the study, of which 314 were in the SfW group and 59 in the control group, meaning that they had both the baseline and the 3 months visits (Figure 1). A total of 278 (89%) of the SfW group participants had a 6 months visit. No 6 months visit was planned for the control group.

Table 1 describes the baseline characteristics. There were statistically significant differences between groups for weight, BMI, SWN total score, 15D and CGI-S total score. A large number of the enrolled persons suffered from schizophrenia, a lesser number from bipolar disorder and other psychiatric diagnoses. More participants in the SfW group had other psychiatric disorders than schizophrenia and bipolar disorder, compared with the control group. The majority of the participants were treated with atypical antipsychotics or antidepressants. Antidepressant medication was about twice as prevalent in the SfW group as in the control group. The mean number of lessons attended by the SfW group was 9.8 (standard deviation: 3.5). A total of 215 (69%) of the SfW participants had 9 or more lessons, 80 (26%) had at least 12 lessons and 9% were registered to have had more than 12. Repeating lessons is allowed in the SfW program.

**Table 1:** Person demographics and illness characteristics at baseline

Person Characteristics	SfW N = 314	Control N = 59	*P-*Values
Female patients, n (%)	180 (57.3)	30 (50.8)	*0.3574*

Age^a^, yrs, (± SD)	41.2 (± 11.97)	39.9 (± 12.55)	*0.4532*

Weight^a^, kg, (± SD)	93.5 (± 20.88)	87.6 (± 18.53)	0.0437*

Height^a^, cm, (± SD)	171.6 (± 9.43)	171.8 (± 9.35)	*0.8765*

BMI^a^, kg/m^2^, (± SD)	31.7 (± 6.48)	29.6 (± 5.33)	0.0173*

Waist circumference^a^, cm, (± SD)	107.5(± 18.52)	105.9 (± 18.90)	*0.5544*

Waist circumference Sensitivity analysis^a^, cm (± SD)	106.4 (± 16.37)	103.5(± 13.89)	*0.2115*

15D total score ^a^, (± SD)	0.814 (± 0.12)	0.857(± 0.09)	0.0085*

SWN total score ^a^, (± SD)	80.5 (± 15.97	87.9 (± 18.35)	0.0016*

**Clinical Characteristics**			

Psychiatric Diagnosis			*0.0627*

Schizophrenia n (%)	163 (51.9)	40 (67.8)	

Bipolar Disorder n (%)	28 (8.9)	5 (8.5)	

Other n (%)	123 (39.2)	14 (23.7)	

Time since diagnosis^a, b^, yrs, (± SD)	9.47 (9.68)	11.36 (12.27)	*0.1911*

CGI-S total score^a^, (± SD)	4.1 (± 0.99)	3.4 (± 1.59)	*< 0.0001*

**Psychotropic medication**			

Atypical antipsychotics n (%) ^c^	203 (64.6)	46 (78.0)	

Antidepressants n (%)	124 (39.5)	12 (20.3)	

Typical Antipsychotics n (%)	70 (22.3)	14 (23.7)	

* = Significant differences between groups ^a ^= mean values, SWN = Subjective Well-being under Neuroleptics Scale, SfW = Solutions for Wellness group, 15D = 15 dimensions quality of life instrument, ^b^N patients = 307 and 59 respectively The most common atypical antipsychotic drugs in the SfW group were olanzapine and risperidone (17% of the patients each), and in the control group the most common atypical antipsychotics were olanzapine and quetiapine (25% and 20% each).

### Impact of the SfW program on subjective well-being and factors associated with response

The rate of participants that had a response (increased score) in the SWN total score at 3 months from baseline was 60.1% for the SfW group and 57.9% for the control group. The between-group difference was 2.2% (95% CI: -11.8; 16.2). From baseline to 6 months, the response rate for the SfW group was 65.5%. After adjustment for potential baseline confounders, participation in the SfW program had no statistically significant impact on response in the initial or sensitivity analysis, neither at Month 3 nor 6 (Table 2). At Months 3 and 6, only the SWN score at baseline was significantly associated with the outcome (lower scores with better outcome), both in initial and sensitivity analysis.

**Table 2:** Baseline factors associated with SWN response (ORs and CI)

Factor	Month 3		Month 6	
	**Initial analysis**	**Sensitivity analysis**	**Initial analysis**	**Sensitivity analysis**

Baseline SWN	0.976 [0.963;0.990]	0.977 [0.963;0.991]	0.963 [0.945;0.980]	0.960 [0.942;0.978]

SfW versus no SfW	0.914 [0.500;1.671]	0.951 [0.516;1.753]	-	-

# Attended Lessons	-	-	0.990 [0.911;1.075]	0.991 [0.911;1.078]

-: not included in the logistic models

### SWN total score mean change throughout the study

The mean change in SWN total score between baseline and 3 months for the SfW group was +3.3 (95% CI: 1.9; 4.7) and for the control group +1.9 (95% CI: -1.2; 4.9). The mean change from 3 months to 6 months was an increase by +1.4 (95% CI: 0; 2.7) for the SfW group.

### SWN subscales: response rates per visit and factors associated with response

In the initial crude analysis, there were statistically significant differences between the SfW group and the control group for two of the five subscales, namely emotional regulation and social integration. After adjusting for baseline covariates and interactions, only the emotional regulation subscale still had a significantly better response rate for SfW participants compared to the control group participants (and this only in the sensitivity analysis) with an odds ratio (OR) of 2.00 (95% CI: 1.05; 3.80).

Other factors associated with response for SWN subscales were the SWN subscale scores at baseline, where lower scores at baseline were associated with a higher chance for response, for all subscales, at both 3 and 6 months, in both initial and sensitivity analysis. These ORs were all in the interval between 0.79 and 0.86 (95% CIs all in the interval between 0.73 and 0.92). For self-control, the duration of illness (measured in years) seemed to have an impact on chance for response at 3 months with OR of 0.97 (95% CI: 0.95; 1.00), there was no difference at 6 months. For physical functioning, higher age (OR: 1.03 [95% CI: 1.01; 1.06]) and shorter duration of illness (OR: 0.95 [95% CI: 0.93; 0.98]) were associated with a higher chance for response at 3 months, but there was no difference at 6 months. For the social integration subscale, higher age was associated with a higher chance for response at 3 months (OR: 1.02 (95% CI: 1.00; 1.04)), but not at 6 months. For social integration, diagnosis (schizophrenia) was found to be a significant factor at month 3 in the initial analysis, but not in the sensitivity analysis, and lower baseline waist circumference seemed slightly associated with an improvement at Month 6, but this was not confirmed by the sensitivity analysis.

### 15D Total score: mean changes throughout study and correlation with SWN scale

From baseline to 3 months the SfW group showed a mean change in the 15D score of 0.023 (95% CI: 0.013; 0.033) and the control group had a mean change of 0.001 (95% CI: -0.016; 0.019). Between 3 months and 6 months the SfW group had a 15D change of 0.002 (95% CI: -0.007; 0.012).

There was a fair degree of concordance between the SWN (total) and the 15D scale: correlations from 0.60 to 0.79.

### Weight changes and associated factors

The mean change in weight from baseline to 3 months was -0.5 kg (95% CI: -0.9;-0.2) for the SfW group and 0.9 kg (95% CI: 0; 1.8) for the control group. Between Month 3 and Month 6, the mean weight of the SfW group decreased by another 0.2 kg (95% CI: -0.6; 0.3).

**Figure 2:**
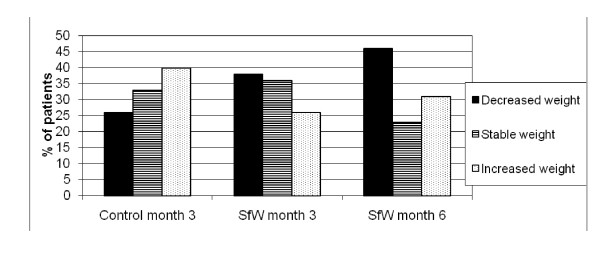
**Weight categorical changes from baseline**. SfW: Solutions for Wellness group.

From baseline to 3 months, 38% had a decrease of their weight, 36% had a stable weight and 26% had an increase of the weight in the SfW group. The corresponding figures for the control group were 26%, 33% and 40% (Figure 2).

Response rates from baseline to 3 months were 74% for SfW participants and 60% for control group participants.

From baseline to 6 months, 46% had a decrease of the weight, 23% had a stable weight, and 31% increased their weight, in the SfW group.

Factors associated with better response at 3 months included participation in the SfW program, older age and being treated in Denmark (Table 3). At 6 months, high baseline SWN scores and living in Denmark or Sweden were found significant.

**Table 3:** Baseline factors associated with weight and waist circumference response (ORs and CI)

Factor	Weight		Waist circumference	
	**3 months**	**6 months**	**3 months**	**6 months**

SfW versus no SfW	1.97 [1.06;3.68]	-	4.96 [2.40;10.34]	-

# Attended Lessons	-	n.s.	-	n.s.

Age (yrs)	1.04 [1.02;1.07]	n.s.	n.s.	n.s.

Norway vs. Denmark	0.44 [0.28;0.85]	0.22 [0.10;0.44]	0.26 [0.12;057]	0.22 [0.09;0.52]

Sweden vs. Denmark	0.46 [0.25;0.86]	n.s.	n.s.	n.s.

SWN total score at baseline	n.s.	1.02 [1.002;1.04]	n.s.	n.s.

Waist circumference at baseline (cm)	n.s.	n.s.	1.185 [*< .0001*]^a^	1.03 [1.005;1.05]

BMI at baseline (kg/m^2^)	n.s.	n.s.	1.215 [0.0964]^a^	n.s.

Waist circ and BMI interaction at baseline (kg/m^2^)	n.s.	n.s.	0.997 [0.0018]^a^	n.s.

a: Type 3 p-value - no confidence interval for OR is provided due to role in interaction

-: not included in the logistic models

None of the factors diagnosis, duration of illness, psychotropic drug at baseline, sex, weight or CGI at baseline had any effect.

### Waist circumference changes and associated factors

In the initial analysis, both the SfW and the control group decreased in waist circumference (WC) between baseline and 3 months, but in the sensitivity analysis the SfW group was observed to have a decrease of the mean waist circumference of 2.2 cm (95% CI: -2.8; -1.6) while the control group had an increase of 1.2 cm (95% CI: 0; 2.4). Between 3 and 6 months, the mean change of the waist circumference was close to zero for the SfW group: -0.1 cm (95% CI: -0.6; 0.5).

**Figure 3:**
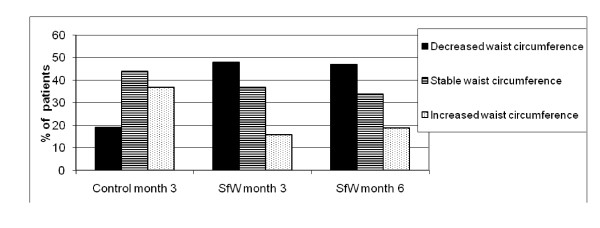
**Waist circumference categorical changes from baseline**. SfW: Solutions for Wellness group.

The rates for decreased, stable and increased waist circumference were 48%, 37% and 16% for the SfW group and 19%, 44% and 37% for the control group from baseline to 3 months (Figure 3).

Response rates from baseline to 3 months were 84% for SfW participants and 63% for control group participants.

From baseline to 6 months, 47% of participants in the SfW group had a decrease of their waist circumference, 34% were stable, and 19% had an increase.

Factors associated with response at 3 months were found to be participation in the SfW program, being in Denmark or Sweden and having a high baseline waist circumference (Table 3). A significant interaction between the latter characteristic and BMI made this impact weaker when associated with a high BMI.

At Month 6, participation in Denmark or Sweden and having a high baseline waist circumference were found to impact the response.

None of the factors age, sex, diagnosis and duration of illness, psychotropic drug at baseline, weight, CGI or SWN at baseline had any effect.

### CGI-S changes over time and associated factors

The mean change from baseline to 3 months was -0.4 (95% CI: -0.5; -0.3) for the SfW group and 0 (95% CI: -0.3; 0.2) for the control group.

The response rates for the SfW and control group from baseline to 3 months were 37.3% and 28.1%. From 3 to 6 months the participants in the SfW group had a change in CGI-S of 0.1 (95% CI: 0; 0.2).

Factors associated with a better response at 3 months were a higher CGI-S at baseline (OR: 1.83, 95% CI: 1.45; 2.29) and a shorter time since diagnosis, in years, (OR: 0.96, 95% CI: 0.93; 0.98). Participation in the SfW program, sex, age, diagnosis, psychotropic drug taken at baseline, country, SWN total score, weight, BMI and waist circumference at baseline did not impact the chance of response.

Factors associated with response at 6 months in SfW participants were: number of attended lessons (OR: 1.3, 95% CI: 1.18; 1.44) and an interaction between country and baseline CGI-S (p = 0.021), though the effect of the country alone (p = 0.0528) and CGI-S alone (p = 0.3472) were hardly or not significant. The country effect seemed to be driven by the difference Sweden vs. Denmark (p = 0.0289, OR = 0.017) rather than Norway vs. Denmark (p = 0.8), suggesting poorer outcomes in Sweden compared to Denmark and similar ones between Norway and Denmark. However, those effects are modified through the interaction with CGI-S at baseline: higher CGI-S score have a positive impact on response in Sweden vs. Denmark (p = 0.0067, OR = 3.34), while none in Norway vs. Denmark (p = 0.319, OR = 1.48).

Sex, age, diagnosis, time since diagnosis, psychotropic drug taken at baseline, waist circumference, BMI, weight and SWN total score at baseline did not have any effect.

## Discussion

The SWN total score response rates for the period between baseline and 3 months were not found to be significantly different for participants who participated in the SfW program and for participants in the control group, neither in the primary analysis nor after controlling for differences in baseline variables. However, for one of the SWN subscales, emotional regulation, OR for response was almost 2 for SfW participants compared with controls in the sensitivity analysis. The content of the emotional regulation subscale is hope for the future, intensity of emotions, interest in what is happening and confidence. Participation in regular group activities for the SfW group participants may have had a positive impact on these aspects. For the other four SWN subscales SfW participation was not associated with a higher chance for improvement of the score.

The only clinical factor associated consistently with improvement in SWN scores was the SWN score at baseline (lower subjective well-being giving a higher probability of improvement). Greater age of the person and shorter duration of illness both were associated with improved subjective well-being on two dimensions each at 3 months: age on physical functioning and social integration and duration of illness on self-control and physical functioning. Diagnosis and symptom severity at baseline were not found to play any role here.

The choice of the short-form of the SWN scale in this study was governed by the history of the scale as it was developed and validated in a clinical trial measuring the subjective well-being of persons with schizophrenia [30], and also because this scale was already part of the SfW program in Scandinavia and the feedback from the SfW instructors about the ability of the participants to fill in the scale was positive.

For the SfW group the mean change from baseline to Month 3 in the SWN total score was +3.3 (95% CI: 1.9; 4.7) and from 3 months to 6 months the mean change was +1.4 (95% CI: 0; 2.7). These changes on SWN score are much smaller than what was reported in another study [34]. That double-blind randomized clinical trial enrolled 114 persons with schizophrenia who changed from other antipsychotics to olanzapine or clozapine. Of the 99 persons who had a SWN score post-baseline, mean improvements in SWN score at 26 weeks (+SD) were found to be 11.3 ± 20.7 points for olanzapine and 8.2 ± 15.8 points for clozapine.

Reasons for observing these differences in change in SWN score may be multiple. Part of the explanation of the much smaller change in SWN score in this study may be that only persons in psychiatric day care or outpatient clinics and inpatients in non-acute wards were included, i.e., relatively clinically stable patients. Only a total of 118 persons of the sample (32%) were markedly ill or more severely affected as measured on the CGI-S scale, compared with the clinical trial where the majority (84% to 93%) was at least markedly ill. Probably, the focus on stable persons in this study makes the evaluation of the impact of participation in SfW on subjective well-being more valid as the effect of other interventions as well as clinical fluctuations over time are smaller for this group than for more acute patients. Another aspect is that the SWN scale, as mentioned, was developed for persons with schizophrenia, and in this study, unlike the trial mentioned above, 170 persons (46%) had another psychiatric disorder than schizophrenia, which means that this scale may not have captured the improvement of all almost half of the persons optimally. Still, the change in QoL as measured with 15D was modest too and not significantly different between the two groups either.

In several important respects, the SfW group differed from the control group, as it had a significantly higher baseline weight and BMI and lower QoL (15D) and subjective well-being (SWN). Furthermore, the SfW group was more severely ill (CGI-S) and contained almost twice as many persons with "other" diagnoses compared to the control group (39.2% vs. 23.7%). The fact that antidepressants were about twice as common in the SfW group (39.6% vs. 20.3%) indicates that depressed persons were substantially more common in the SfW group. Persons with depression may have more pronounced difficulties finding the motivation necessary for any change in lifestyle, both regarding diet changes or physical exercise habits, which has possibly contributed to the modest change in SWN score in the SfW group. Most of the above mentioned differences between the two groups were controlled for in the logistic regression analyses (weight, BMI, SWN and CGI-S at baseline), but as diagnoses were not collected for the persons who did not have either schizophrenia or bipolar disorder (i.e. they were categorized as "other") it was not possible to adjust fully for the differences in diagnoses between SfW participants and controls. The reason for the way diagnoses were registered in the study (schizophrenia/bipolar disorder/other) is that the SfW program was started with persons with schizophrenia in mind, and it was expected that almost all participants would have schizophrenia (which was also the reason for choosing the SWN scale as an outcome measure). However, this study has shown that the program is now used for a much more diverse population. The differences in diagnoses between the two groups is a limitation of this study (due to its non-randomized design), and likewise, there may also be other variables that influenced the outcome, which were not equally distributed between the SfW participants and the controls, and which were not measured, and which could therefore not be controlled for in the statistical analysis. Examples of such variables could be: previous weight gain, eating disorders and treatment changes during or after the program. These differences between the two groups, both the ones that are known now and the ones that may exist, but were not investigated as we wanted to keep the study simple, illustrate the main limitation of using a non-randomized design, which is the difficulty of assessing the validity of differences found, or not found, between the groups in terms of outcome of the interventions.

As described, the control group consisted of persons eligible for entering the SfW program but treated at hospital departments and outpatient clinics which did not run the SfW program. This design was chosen in order to ensure a clear separation between the SfW group and the control group in order to be able to investigate the relationship between participation in SfW and any observed change. However, whereas the treatment of the psychiatric disorders must be assumed to be comparable in centers running the SfW program and centers not running the SfW program, it was out of our control if the centers collecting participants for the control group had other programs or activities with a similar effect on the well-being as the SfW program. In theory, a better design could be to run a cluster-randomized clinical trial, randomizing departments and clinics to program participation or not, but at the time when it was decided to run this study the SfW program was already established in many settings in Scandinavia.

Alternative designs would have been to recruit persons for the SfW group and for the control group in the same departments and clinics, with or without randomization to the two groups, so that the participants in the two groups would be exposed to the same treatment of psychiatric disorders and the same "other activities", but this was found suboptimal as the care of the participants in the control group would be influenced by the fact that the program was run by the department, as the change in knowledge and attitudes induced among the caregivers through working with the program would have an impact on all patients. Still, having decided to recruit SfW patients and control patients in different departments, it could have been possible to explore and describe the differences between SfW centers and the control centers in terms of what "other activities" they were running in order to try to evaluate the possible impact of these, and using matched controls instead of controls who "only" were eligible for participation in the program could have reduced the differences between the groups and improved the validity of the results.

The country where the study participants were enrolled was significantly associated with the probability of response for both weight and waist circumference at both time points: participants in Denmark had better chances to stabilize their weight at Month 3, and having participated in the study in Denmark or Sweden was associated with a better outcome for weight at Month 6 and for waist circumference at both time points. The results presented in this publication are mean results achieved across centers with different ways of implementing the SfW program and working with lifestyle intervention in general. The differences regarding weight and waist circumference control between the three countries suggest that different approaches were developed at a country level. The way the program was implemented was not registered as part of the study, but differences between centers were noticed: some centers focused on diet, others on physical activity, some taught mainly theory, whereas others used a more active and practical approach with for example outdoor activities. The SfW as an educational program is focusing on knowledge and attitudes. But it is not increased knowledge that changes the weight, but improved lifestyle habits. A more practical approach, perhaps leading to a larger degree of change of behavior among the participants, might have generated a larger mean weight loss. The differences in results between centers with different approaches have not been analyzed in this piece of work, but would be an interesting next step for a research project.

A number of other studies evaluated the impact on weight of behavioural programs more or less similar to the Scandinavian SfW program, and the results from these studies are generally in line with the findings from the study reported here with regards to development during program participation [9-28]. Some of these studies also followed the participants after program cessation to evaluate the persistence of the effect on weight [9,18,20,21], and these studies found, like the one reported here, that the effect of the program seemed to last in the period from the end of the program and up to the end of follow-up a few months later. It is interesting to see how the results from this study and many other studies match in this respect, and it is important to acknowledge that even though the weight loss during SfW program participation was not large, 0.5 kg, the persons were referred to the SfW program because of gaining weight or having weight problems, and while participating in the program 38% of the persons lost weight and 36% stayed stable. These, findings, all pointing in the same direction, contrast with the results on subjective well-being and QoL. Only a few studies included these outcome measures, and among three randomized controlled trials only one found a positive impact of the intervention on QoL [10,18,24]. However, all these studies had small sample sizes, and low statistical power may have contributed to the lack of finding of positive changes in QoL in the other two studies.

The SfW group was found to improve significantly from baseline to Month 3 regarding the severity of the psychiatric disorder as measured with CGI-S score, whereas the control group did not change. When interpreting this finding it is relevant to take in consideration that the study was an observational, open-label study, and that bias may play a role for this difference between the groups. Some centers made CGI-S ratings at clinical conferences, where staff not working with SfW was also present. This is a good practice in order to reduce the bias introduced when investigators rate their own patients and through that the quality of their own work. This practice was, however, not implemented in all centers.

The statistical methods used illustrate the exploratory nature of the study. No clinically meaningful difference has been defined for the primary outcome measure, the SWN scale, and it was decided to use a difference in the proportion of participants with a SWN improvement between the two groups of 70% - 50% or larger for the sample size calculation. In order to reach a power of 80%, with a two-sided type I error of 0.05, 300 persons were enrolled in the treatment group and 55 persons in the control group. Uneven treatment groups were chosen as the widespread implementation of the SfW program would have made recruitment of more SfW-naïve departments and clinics difficult.

## Conclusion

In conclusion, the Solutions for Wellness program was not shown to have a positive impact on the subjective well-being of the participants relative to the control group, as measured with the SWN scale, but Solution for Wellness program participation was associated with maintaining weight and waist circumference and the program may allow health care professionals to help persons under psychotropic treatment to control their weight.

## Competing interests

VP, OKK and PS are employees of Eli Lilly and Company. HN and CB are former employees of Eli Lilly and Company. EWM and EL have no competing interests.

## Authors' contributions

VP conceived of the study, participated in design of the study, was involved in interpreting the data and drafted the manuscript. CB was responsible for statistical analyses, was involved in interpreting the data and revised the manuscript. OKK conceived of the study, participated in design of the study, participated in coordination of the study, was involved in interpreting the data and revised the manuscript. EWM and EL were involved in interpreting the data and revised the manuscript. HN was involved in interpreting the data and drafted the manuscript. PS conceived of the study, participated in design of the study, was involved in interpreting the data and revised the manuscript. All authors read and approved the final manuscript.

## Pre-publication history

The pre-publication history for this paper can be accessed here:

http://www.biomedcentral.com/1471-244X/10/42/prepub
